# An active DNA-based nanoprobe for photoacoustic pH imaging†
†Electronic supplementary information (ESI) available: DNA sequences, optical characterisation and calculation details, Fig. S1–S8 and Tables S1, S2. See DOI: 10.1039/c8cc04007a


**DOI:** 10.1039/c8cc04007a

**Published:** 2018-08-23

**Authors:** Kevin N. Baumann, Alexandra C. Fux, James Joseph, Sarah E. Bohndiek, Silvia Hernández-Ainsa

**Affiliations:** a Cavendish Laboratory , Department of Physics , University of Cambridge , Cambridge , UK . Email: seb53@cam.ac.uk; b Cancer Research UK Cambridge Institute , University of Cambridge , Cambridge , UK; c Instituto de Nanociencia de Aragón (INA) , University of Zaragoza , Campus Río Ebro, Edificio I+D , 50018 Zaragoza , Spain . Email: silviamh83@unizar.es; d ARAID Foundation , Government of Aragon , Zaragoza 50018 , Spain

## Abstract

We report an active DNA construct capable of probing pH through a photoacoustic (PA) ratiometric analysis approach. Our nanoprobe enables different PA readout in tissue mimicking phantoms in the range between pH 6.8 to 7.8 at physiologically relevant sodium concentrations. Thus, it represents a promising platform to probe pH values relevant to the tumor microenvironment using PA.

## Abstract 2

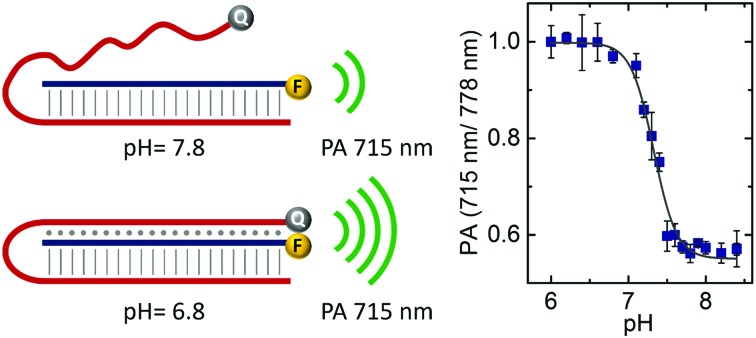
A DNA-based nanoprobe sensitive to pH has been developed for pH photoacoustics imaging through a ratiometric approach.

## 

DNA nanotechnology offers numerous possibilities for the creation of stimuli-responsive nanomaterials.^1^ This includes the incorporation of responsive molecules into the DNA framework to establish activatable nanoplatforms whose responses can be controlled using light^2^,^3^ or voltage.^4^ Unmodified oligonucleotides are also capable of yielding stimuli-responsive DNA structures upon appropriate selection of sequences that can be triggered by several chemical inputs including pH.^5^–^7^ Such pH responsive DNA nanostructures have recently attracted much interest in nanomedicine as nanocarriers, for pH-controlled release of drugs,^8^ or as nanoprobes capable of tracking pH changes *in vitro*^9^,^10^ and *in vivo*.^11^ Monitoring the alteration of intracellular and/or extracellular pH *in vivo* can aid the diagnosis and prognosis of several diseases, including cancer.^12^ Most of these pH-sensitive DNA-based nanostructures consist of nanostructures decorated with selected fluorophore pairs that exhibit changes in resonance energy transfer in response to changes in pH levels and are often monitored recording their fluorescence emission levels.^6^,^7^,^9^,^10^,^13^ Using these all-optical fluorescence measurements results in limited penetration depth and spatial localisation ability due to strong scattering of light in biological tissues.^14^ However, with the advent of photoacoustic imaging (PAI), it is now possible to obtain high resolution optical images from greater depths in tissue and to therefore overcome these traditional limitations of all-optical imaging.

In this communication we present a pH-sensitive DNA-based nanoprobe capable of generating a specific PA signal readout and validate its potential capabilities for future *in vivo* imaging using tissue mimicking phantoms. Conventionally, PAI of pH has been previously achieved by activatable dyes working at fixed pH ranges.^15^–^18^ Our approach however, enables the design of a nanoprobe to act at a desired pH range through precise DNA sequence selection.^6^ Specifically, our pH nanoprobe was designed to act in the range of pH 6.0 to 8.0, which is relevant for tumour imaging applications.^12^,^19^ The nanoprobe is composed of two strands hybridised *via* Watson–Crick base pair interactions over a 21nts-long domain, with a non-hybridised 26nts-long single-stranded domain capable of forming an intramolecular triplex through Hoogsteen base-pair interactions (Fig. 1a). The strands are either homopurine or homopyrimidine to enable the formation of CGC triplets upon protonation of the cytosine, which promotes the formation of the triplex.^20^ The triplex is further stabilised by TAT triplets thereby enabling our DNA nanoprobes to be responsive to changes in pH levels. A 5-bases loop was introduced in the nanoprobe to allow its folding into a triplex based on previous designs by Ricci and collaborators^6^ (sequences are gathered in Table S1, ESI†).

**Fig. 1:**
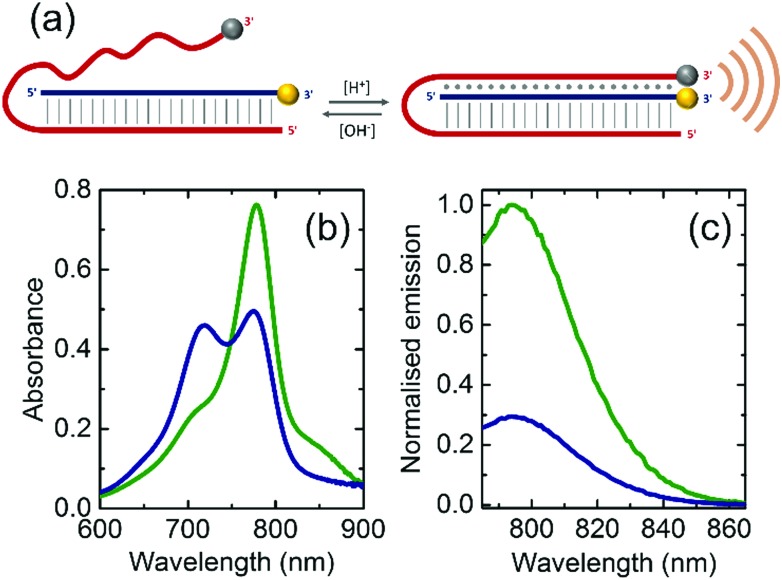
(a) Scheme of the pH-sensitive DNA nanoprobe. Vertical lines represent the domain of Watson–Crick base pairing hybridisation and dots show the domain of Hoogsteen interactions. IR800CW Dye (F) and IRQC1 Dye (Q) are shown as yellow and grey spheres respectively. Orange waves represent the increase in PA signal due to the quenching between F and Q. (b) Absorbance spectra and (c) normalised emission spectra of the DNA nanoprobe at pH = 6.8 (green curve) and pH = 7.8 (blue curve) in a buffer containing 150 mM Na^+^ at 34 °C.

We selected the strands then decorated at their 3′ position with the IR800CW Dye (fluorophore, F) and the IRQC1 Dye (quencher, Q) as PA reporters (see ESI†). This selection was based on our recent demonstration of the capabilities of this F/Q pair to produce contact quenching when positioned in close proximity. Their relative placement renders a change in the absorbance spectrum compared to the free F and Q, and consequently a modification in the wavelength-dependent PA response.^21^

The pH-responsive nanoprobe was folded at 2 μM equimolar DNA concentration in a solution containing 150 mM Na^+^ with a phosphate buffer (pH = 7.2) using a thermal gradient and characterised by polyacrylamide gel electrophoresis (PAGE) (see Fig. S1 and ESI† for further details).

Upon folding, the pH levels of the solution that contained the nanoprobe was adjusted either to pH = 6.8 to induce triplex formation or to pH = 7.8 to disrupt it. The absorbance spectrum at pH = 7.8 shows a maximum peak at 778 nm originated by the IR800CW Dye as well as a shoulder centred at 830 nm corresponding to the IRQC1 Dye (green curve in Fig. 1b). On the contrary, the spectrum at pH = 6.8 shows a distinctive double peak with the absorption maxima at 719 and 778 nm (blue curve in Fig. 1b), originated from a contact quenching process between F and Q due to triplex formation.^21^,^22^ The triplex formation is also evidenced by a decrease in the fluorescence signal of the IR800CW Dye at lower pH as given by the fluorescence emission measurements (Fig. 1c).

A gradual increase in the intensity of the peak centered at 719 nm as well as a decrease of the intensity maximum at 778 nm was observed while the pH was decreased from 7.8 to 6.3 (Fig. S2, ESI†). We quantified this change in the absorbance spectra by applying a ratiometric analysis^23^,^24^ calculating the ratio of the absorbance intensities at 719 nm and 778 nm. The ratiometric values were then plotted against their corresponding pH levels. The experimental data were fitted with a Boltzmann fit to approximate the pH at which the transition of this ratio occurs. This pH transition value validates the range over which our nanoprobe is capable of distinguishing different pH levels and is related to the triplex-to-duplex transition. Reversibility of this pH-driven transition was proved by absorbance measurements (see Fig. S3, ESI†). This transition was investigated in samples prepared at three different concentrations of Na^+^. As observed in Fig. 2, a slight increase of the transition pH value was observed as the Na^+^ concentration was increased from 50 to 450 mM (pH = 7.09 ± 0.02 at 50 mM, pH = 7.23 ± 0.02 at 150 mM and pH = 7.46 ± 0.03 at 450 mM). This indicates that at this studied range of concentrations, Na^+^ stabilises the closed state of the nanoprobe (see Fig. S4, ESI†). Thus, the Na^+^ concentration is important to be considered for an accurate pH determination with the nanoprobe.

**Fig. 2:**
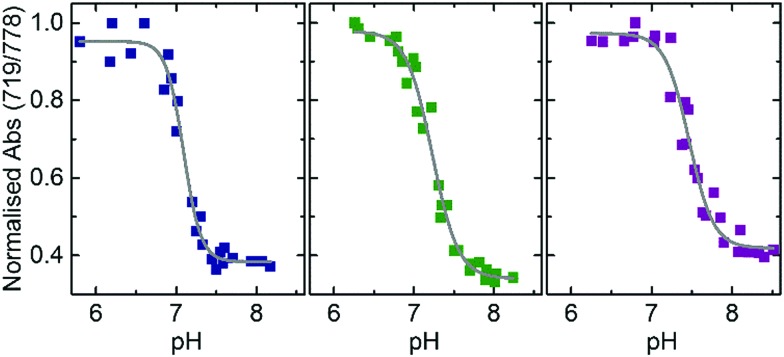
Absorbance ratio (719/778 nm) *versus* pH given by the DNA nanoprobe in a buffer containing different concentrations of Na^+^ (50 mM, blue data), (150 mM, green data) and (450 mM, purple data). Each salt concentration condition contains at least 23 data points that correspond to samples prepared in four independent batches. Grey lines represent the Boltzmann fitting to the data. *r*^2^ values resulting from the fitting are: 0.98, 0.98 and 0.96 for the buffers containing 50 mM, 150 mM and 450 mM of Na^+^ respectively.

Once we demonstrated the use of our nanoprobe for a ratiometric absorbance detection of pH, we further studied the pH dependent PA signal generation capabilities of the nanoprobe under tissue mimicking conditions.

DNA nanoprobes were encapsulated inside thin-walled transparent tubes located at 1 cm depth in the center of cylindrical tissue mimicking phantoms, prepared as described previously.^21^ PAI of the samples was then achieved using a commercial imaging system, using methods that were validated previously.^25^ Multispectral PA signals were acquired from 4 separately prepared sets of nanoprobes covering a range of pH values from 6.0 to 8.4 measured at three different scan positions along the phantom. Quantification of the PA response was performed by extracting the mean pixel intensity (MPI) values from a region of interest (ROI) drawn within the straw position in the reconstructed images (see Section S4 and Fig. S5, ESI†). Similar to the optical absorbance spectra, the obtained PA spectrum at pH = 6.8 shows higher intensity at lower wavelengths (peak at 715 nm) than at pH = 7.8 (compare blue and green data in Fig. 3a).

**Fig. 3:**
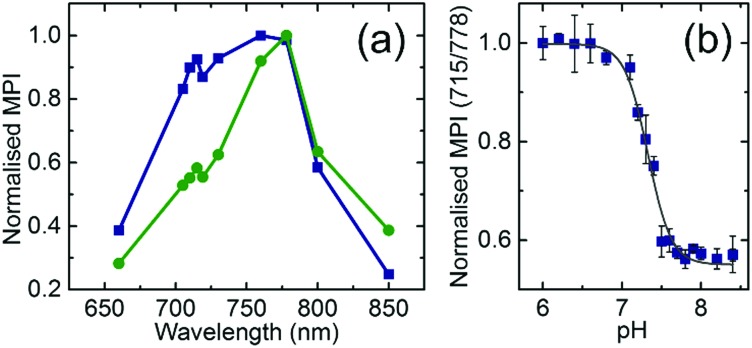
(a) Photoacoustic response derived from the mean pixel intensity (MPI) value extracted from a region of interest (ROI) at different wavelengths for the DNA nanoprobe in a buffer containing 150 mM Na^+^ at pH = 6.8 (blue curve) and pH = 7.8 (green curve) at 34 °C. (b) Ratiometric PA pH imaging of phantoms. Ratio of the MPI values extracted from the ROI at 715 nm and 778 nm calculated in phantoms containing the DNA nanoprobe in a buffer containing 150 mM Na^+^ at different pH values. Data of 4 independent sets are included. Error bars represent the error propagation of the standard deviation, calculated with the Taylor propagation. The grey line represents the Boltzmann fitting to the data. The *r*^2^ value resulting from the fitting is 0.99.

The ratiometric PA dependence of the pH was analysed similarly to the absorbance case (Fig. 3b). The Boltzmann fit of the PA measurement data reveals a transition at pH = 7.31 ± 0.02 which is in good agreement with the obtained values for absorbance at that salt concentration.

In conclusion, we have demonstrated the use of a pH-sensitive DNA nanostructure to probe pH through a ratiometric PA analysis approach. In particular, our nanoprobe enables pH values to be distinguished in the range of pH 6.8 to 7.8, which is relevant for the physiological range and could be used to assess pH differences exhibited by healthy tissues and the tumour microenvironment.^12^,^19^ The working pH range of our DNA nanoprobe is also dependent on sodium concentrations. We validated the suitable range for a buffer containing 150 mM Na^+^, which approximates to biological conditions. It is expected that upon integration into biologically stable nanocarriers, the reported nanoprobe could be a powerful tool for cancer diagnosis and prognosis using non-invasive PAI.

This work was funded by a Cancer Research UK Cambridge Centre Pump Prime Research Grant. K. N. B. and A. C. F. acknowledge the ERASMUS placement organisation for ERASMUS+ funding. K. N. B. acknowledges the DAAD for a PROMOS scholarship. J. J. and S. E. B. are funded by the EPSRC-CRUK Cancer Imaging Centre in Cambridge and Manchester (C197/A16465); CRUK (C14303/A17197, C47594/A16267) and the EU-FP7-agreement FP7-PEOPLE-2013-CIG-630729. S. H. A. acknowledges funding by the University of Zaragoza (UZ2018-CIE-04) and the support of ARAID.

## Conflicts of interest

There are no conflicts to declare.

## Supplementary Material

Supplementary information
