# Supplementary material for: An active DNA-based nanoprobe for photoacoustic pH imaging
Source: Chem Commun (Camb). 2018 Aug 23;54(72):10176–8. doi: 10.1039/c8cc04007a (PMC6127833; doi:10.1039/c8cc04007a)
Supplement: Supplementary file 1 [file CC-054-C8CC04007A-s001.pdf]

Electronic Supplementary Material (ESI) for ChemComm.
This journal is © The Royal Society of Chemistry 2018

Supporting Information

An active DNA-based nanoprobe for photoacoustic pH imaging

Contents:

S1. Assembly and characterisation of the DNA nanoprobe

S2. Absorbance and emission measurements and calculations

S3. pH reversibility of the DNA nanoprobe

S4. Photoacoustic imaging and data quantification

S5. pH determination in other buffers

S6. Stability of the DNA nanoprobe

Supporting Figures S1-S8

Supporting Tables S1, S2

S1. Assembly and characterisation of the DNA nanoprobe

The DNA strands were purchased from IDT (Integrated DNA Technologies, Inc). Sequences are collected in table S1. Aliquots were stored in the freezer protected from light. Oligos were mixed in a 1:1 ratio to a final concentration of 2μM in the corresponding phosphate buffer. Buffers were prepared with Na_2HPO_4 at either 25mM, 75mM or 225mM to render 50mM, 150mM or 450mM of Na^+ respectively. pH was adjusted with the minimum volume of NaOH (4M) or HCl (32%). The assembly was carried out in a thermocycler using the following thermal-annealing: From 70 to 25°C in 90 steps (0.5°C per step, 30 s each step) and the synthesized samples were stored at 4°C. [1]

Correct assembly was assessed with polyacrylamide gel electrophoresis (PAGE) (figure S1). Samples were run in PAGE (10%) at 100 V for 40 min in a solution containing 11mM $MgCl_2$ buffered with 0.5xTBE (pH = 8.3). Low molecular weight DNA Ladder (Invitrogen, Thermo Fisher Scientific Inc.) was used as reference. The gels were stained with GelRed for 5 minutes and visualized using a Syngene Gel Doc system.

S2. Absorbance and emission measurements and calculations

The absorbance and fluorescence emission of the DNA nanoprobes at the corresponding pH were measured at 2µM of DNA concentration. Data were collected at 34°C in order to mimic photoacoustic measurement temperature conditions. pH was adjusted with the minimum volume of NaOH (4M) or HCl (32%). Absorbance change as a function of pH is shown in figure S2. The ratiometric analysis shown in the main text was performed as follows. Initially, the value resulting from the ratio of the intensities of the absorbance spectra at 778 and 719 nm was calculated for each sample (studied at each pH condition). Then this value was plotted as a function of pH. The graph of the ratio against pH was then fitted using a Boltzmann fit using OriginPro2016 to obtain the pH transition and its associated standard error (given by the fit). DNA melting curves were taken by recording the absorbance of the samples at 260 nm applying at a rate of 1°C/min. The derivatives of the melting curve data were calculated using OriginPro2016 to obtain the melting transitions (T1 and T2). T1 and T2 values correspond to the maxima of the derivative curves (resulting from the Gaussian fitting of the curves with OriginPro2016). T1 and T2 dependence with pH for the DNA nanoprobes folded at different [Na^+] are collected in figure S4. T1 occurs due to the transition of closed to open triplex (rupture of Hoogsteen interactions) and hence it increases at lower pH since the amount of triplex formed at low pH is larger. T2 corresponds to the transition of closed to open duplex domain.[2] Absorbance was measured using a UV-Vis Spectrophotometer (Varian Cary 300 Bio, Agilent Technologies, Inc.)

For emission measurements, fluorescence excitation was set to 778 nm, which corresponds to the absorbance maxima of the unfolded triplex. Emission data were collected with a Fluorescence Spectrophotometer (Varian Cary Eclipse, Agilent Technologies, Inc.).

S3. pH reversibility of the DNA nanoprobe

The pH reversibility of the DNA nanoprobe was investigated with absorbance measurements. The reversible pH response of the nanoprobe was proved in 3 cycles of subsequent pH modification. This pH change was performed with the addition of either NaOH (4M) or HCl (32%). Figure S3 gathers the absorbance spectra collected at each cycle.

S4. Photoacoustic imaging and data quantification

Photoacoustic imaging of the samples was performed using a commercial photoacoustic tomography (PAT) system (inVision256-TF; iThera Medical GmbH) and tissue mimicking phantoms. The commercial PAT system has been described and characterised previously[3] . It uses a tunable (660-1300 nm) optical parametric oscillator pumped by a nanosecond pulsed Nd:YAG laser operating at 10 Hz repetition rate. The phantom was mounted onto the sample holder and was translated in the z-direction with a step size of 1 mm, 10 time frames averaging across 3 distinct scan locations. Photoacoustic signals were generated at specific excitation wavelengths (660, 705, 710, 715, 719, 730, 760, 778, 800, 850 nm) and detected using 256 toroidally focused ultrasound transducers operating at 5 MHz center frequency and 60% bandwidth. The images were reconstructed using the system integrated algorithm.

The tissue phantoms were fabricated with the optical and acoustic properties of biological tissues as reported previously.[3] Briefly, the phantoms were fabricated using agar as the base material; nigrosin dye and intralipid were added to provide an absorption coefficient of 0.05 cm^{-1} and reduced scattering coefficient of 5 cm^{-1}. The DNA nanostructures were held inside sealed thin walled plastic tubes (0.3 cm diameter) that were placed at the center of the cylindrical phantoms (2 cm diameter). For all the measurements, the phantoms were maintained at 34°C inside the water bath.

The pH-dependent transition point was analysed by a ratiometric approach. The photoacoustic mean pixel intensities (averaged over 3 slice positions) at 715 nm and 778 nm excitation (I_{715} and I_{778}, corresponding to the two maxima found in the PA spectrum) were divided by each other to obtain the peak ratio (PR):

$$PR(I_{715}, I_{7785}) = \frac{I_{715}}{I_{778}}$$

PR errors were obtained by calculating the error propagation with the variance formula:

$$total\ error = \sqrt{\left(\frac{\partial PR}{\partial I_{715}}\right)^2 \sigma_{I,715}^2 + \left(\frac{\partial PR}{\partial I_{778}}\right)^2 \sigma_{I,778}^2}$$

With:

$\frac{\partial PR}{\partial I_{715}} = \frac{1}{I_{778}}$ derivative with respect to the I_{715} value

$\frac{\partial PR}{\partial I_{778}} = -\frac{I_{715}}{{I_{778}}^2}$ derivative with respect to the I_{778} value

$\sigma_{I,715} = \sigma_{I,778} =$ standard deviation of the I_{715} and I_{778} values respectively

For more than one sample per pH value, the overall deviation was pooled adding the total errors (as calculated before), normalised by the number of samples per pH:

$$pooled\ total\ error = \sqrt{\frac{\sum_i^n total\ error_i^2}{n}}$$

Finally, the values obtained by these calculations (peak ratio and total error) were normalised to the maximum peak ratio set to 1.

S5. pH determination in other buffers

The capabilities of the DNA nanoprobe to detect pH were investigated in Tris (50mM) with 150mM NaCl and PBS. DNA nanoprobes were prepared at 2µM DNA concentration in these buffers (3 samples per buffer) and the absorbance spectra were collected (see figure S6). Similarly, PAT images were collected for these nanoprobes in the different assessed buffers (see figure S7).

A ratiometric analysis was then performed as described in section S2 and S4. The pH of the solutions given by the nanoprobe was calculated using the Boltzmann curves obtained from the fitting of the data shown in figure 2 (green data) and 3b of the main text. As observed in table S2, calculated pHs are in line with the measured pH of the buffers.

S6. Stability of the DNA nanoprobe

The stability of the DNA nanoprobe was investigated during a week. Absorbance was measured just after the folding of the DNA nanoprobe and at different time points during a week after folding. During this period, sample was stored at 4°C and protected from light. Absorbance measurements were performed at 34°C. As observed in Figure S8, the absorbance spectra remain similar after one week of incubation which indicates that under the assessed conditions nanoprobes can be used at least 1 week from preparation.

Table S1. Sequences of the oligos used for the pH nanoprobe

**Oligos**	**Sequence**
Quencher oligo	TCCCTTTCCCTTTTTCTTCTTCTTTGTTCTTCTTTTTCCCTTTCCCT/IRQ Dye QC-1/
Fluorophore oligo	AAGAAGAAAAAGGGAAAGGGA/IRDye 800CW/

Table S2. Measured and calculated pH values in PBS and Tris

Buffer	*Measured pH at 34°C*	*Estimated pH via abs ratiometric analysis*	*Estimated pH via PAT ratiometric analysis*
PBS	7.3	7.34±0.03	7.24±0.04
Tris	7.4	7.46±0.01	7.38±0.03

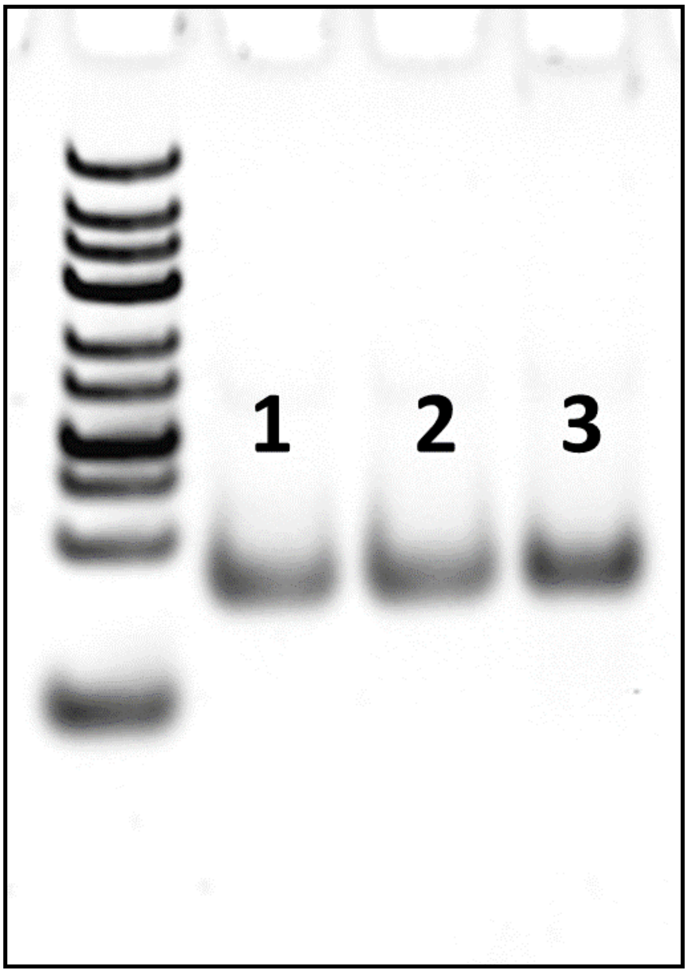

Figure S1. PAGE gel of DNA nanoprobes folded at 2µM DNA concentration in a solution containing 50mM (lane labelled as 1), 150mM (lane labelled as 2) or 450mM (lane labelled as 3) [Na^+] in a phosphate buffer (pH=7.2). Each nanostructure resolves into a single band. DNA ladder (Low Molecular Weight, NEB) is included.

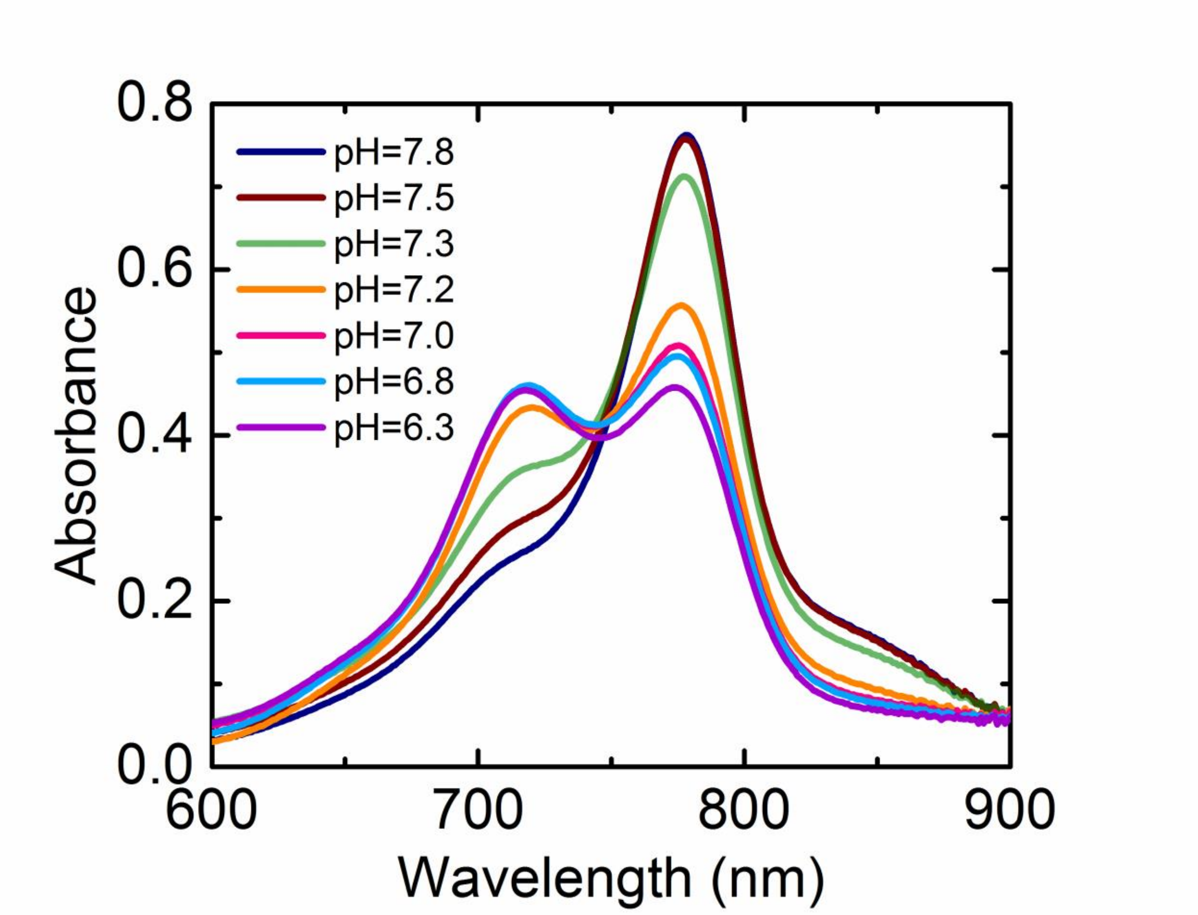

Figure S2. Absorbance spectra of the DNA nanoprobe at 2µM concentration in 150mM [Na^+] solution buffered at different pHs.

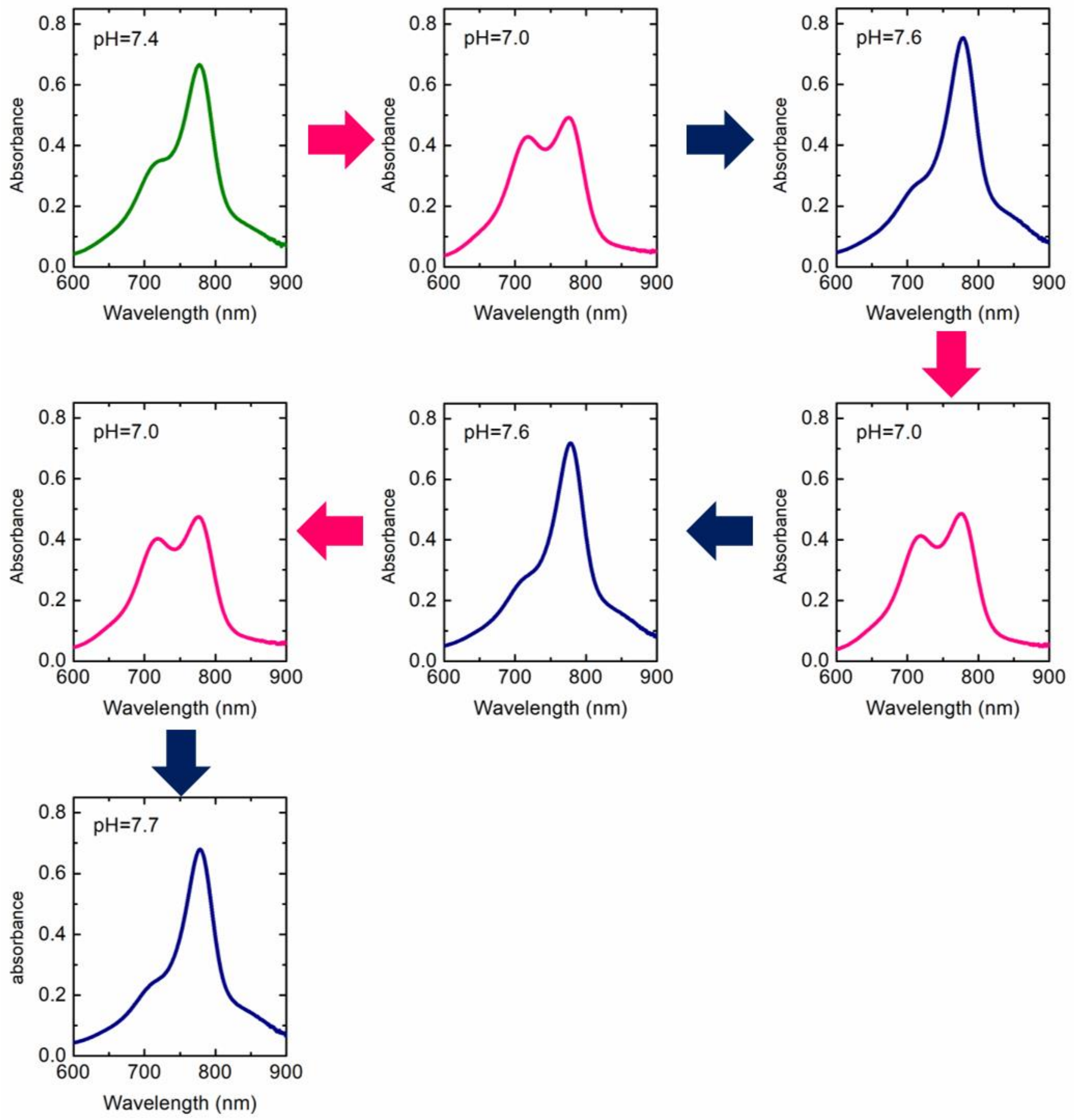

Figure S3. Absorbance spectra of the DNA nanoprobe (2μM) in 75mM Na_2HPO_4 buffer at subsequent decrease and increase of pH. The absorbance spectrum of the initial sample is shown in green. Pink and blue arrows indicate HCl and NaOH addition respectively. The absorbance spectra of the samples after pH change are shown in pink (after HCl addition) and blue (after NaOH addition).

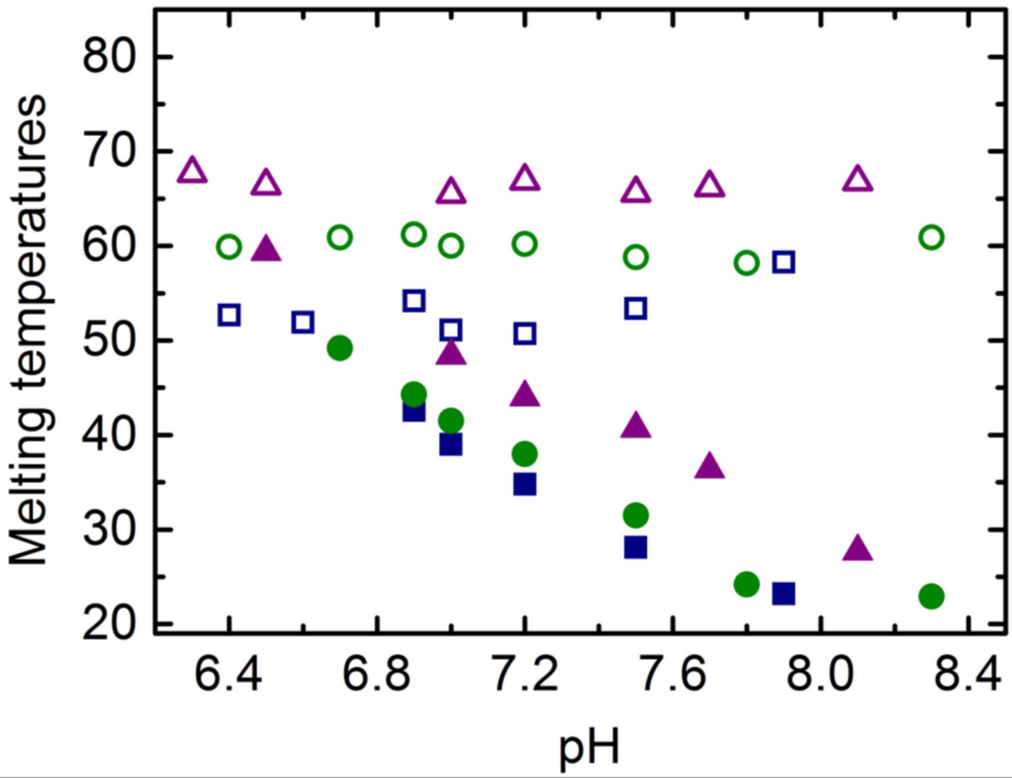

Figure S4. Melting temperatures of the DNA nanoprobes at different pHs and different Na^+ concentrations. T1 values are shown as full symbols. T2 values are shown as empty symbols. Purple, green and blue data represent different [Na^+], namely 450, 150 and 50mM respectively. At sufficiently low pHs (data at pH<6.9 for 50mM [Na^+], pH<6.7 for 150mM [Na^+] and pH< 6.3 for 450mM [Na^+]) where all the DNA nanoprobes are present as closed triplex only one melting temperature is observed corresponding to the direct transition from triplex to single strands.

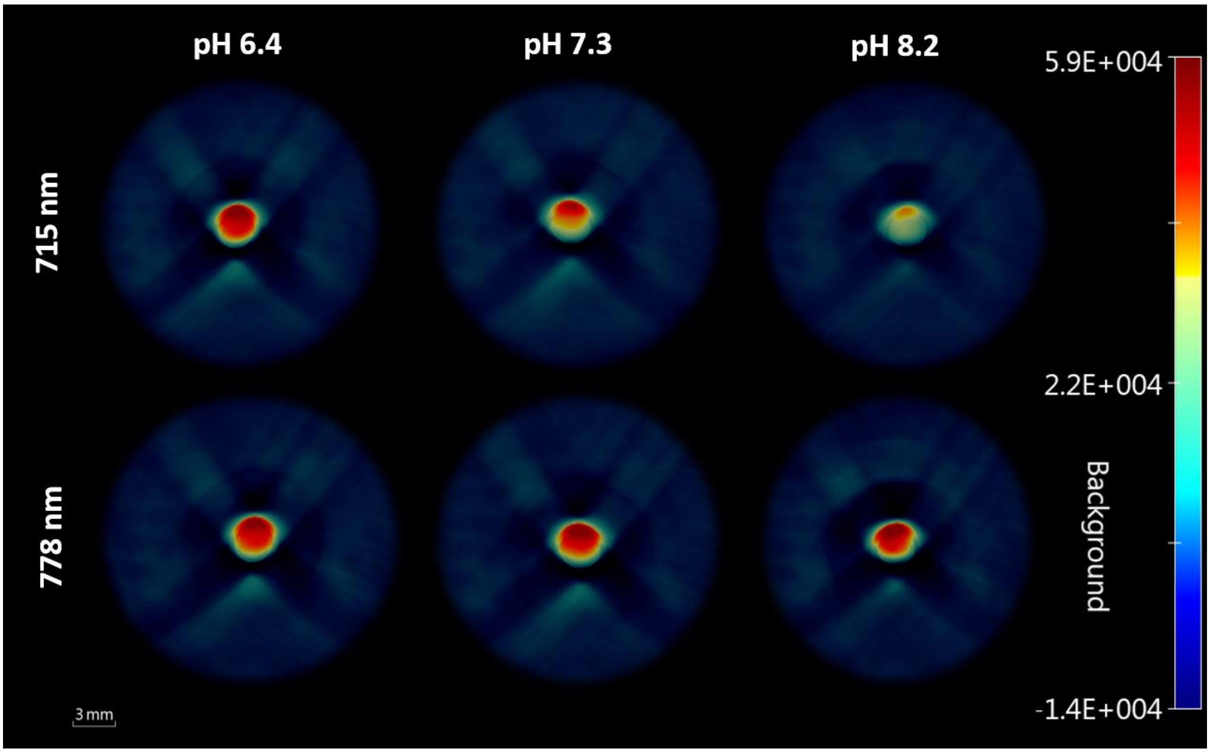

Figure S5. Photoacoustic images of DNA nanostructures at 715 nm and 778 nm excitation wavelengths. DNA nanostructures with different pH levels were held inside the tissue mimicking phantoms.

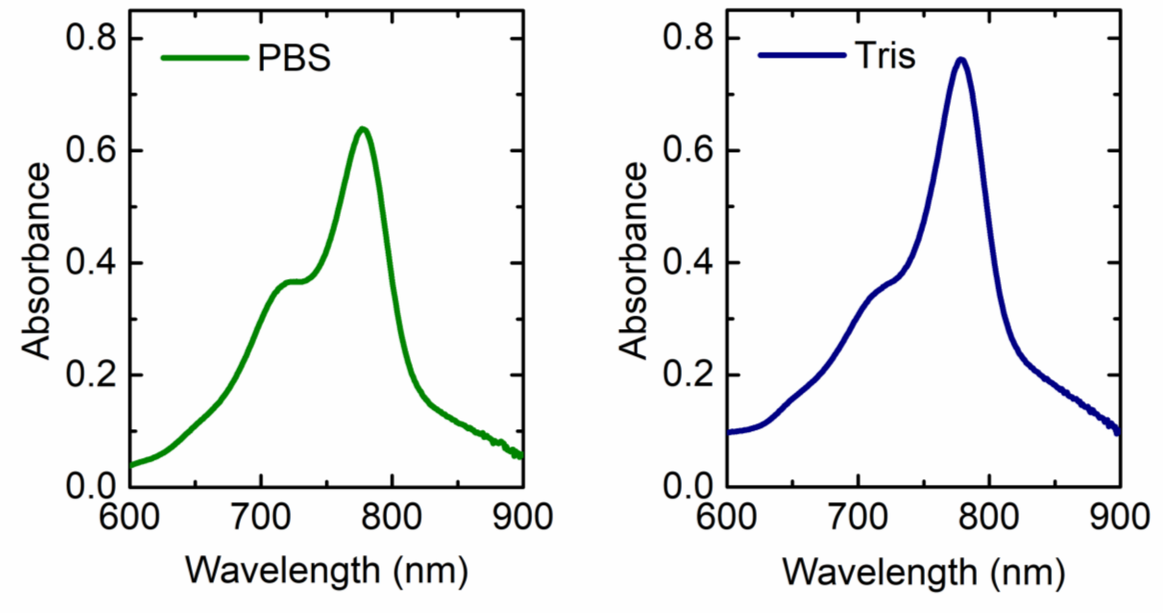

Figure S6. Absorbance spectra of the DNA nanoprobes in PBS and Tris.

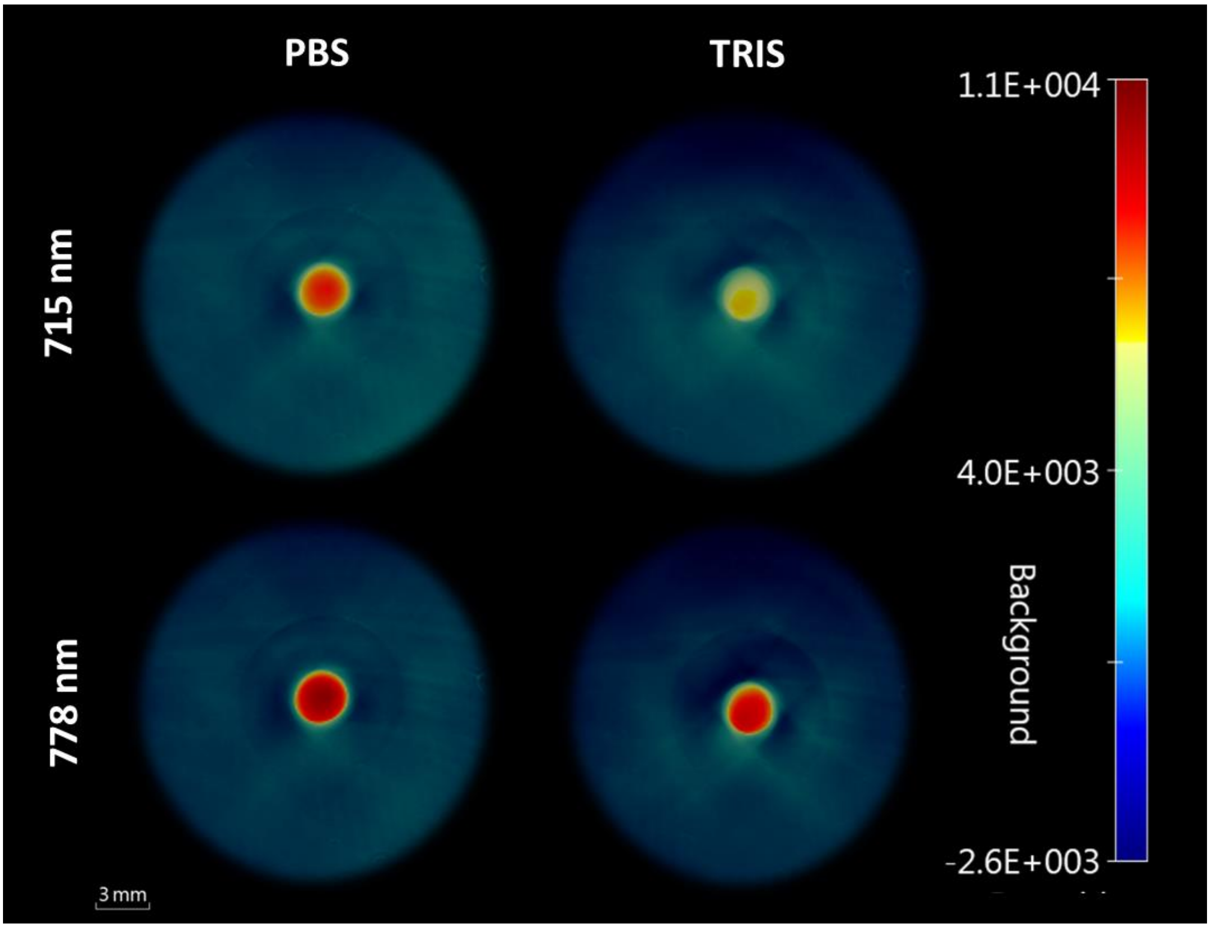

Figure S7. Photoacoustic images of DNA nanostructures at 715 nm and 778 nm excitation wavelengths in PBS and Tris.

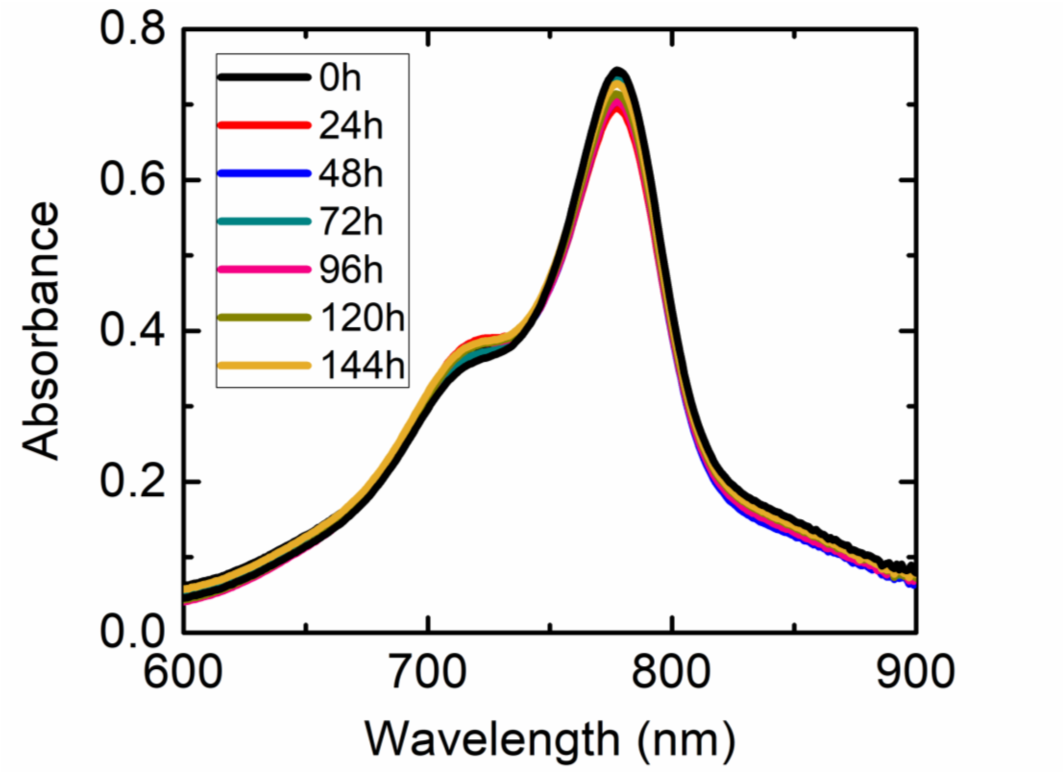

Figure S8. Absorbance spectra of the DNA nanoprobe in 75mM Na_2HPO_4 buffer at pH=7.4 collected at different time points over a week.

REFERENCES

[1] J. Joseph, K. N. Baumann, P. Koehler, T. J. Zuehlsdorff, D. J. Cole, J. Weber, S. E. Bohndiek and S. Hernández-Ainsa, *Nanoscale*, **2017**, *9*, 16193-16199

[2] M. Mills, P. B. Arimondo, L. Lacroix, T. Garestier, H. Klump and J. L. Mergny. *Biochemistry* **2002**, *41*, 357-366

[3] J. Joseph, M. R. Tomaszewski, I. Quiros-Gonzalez, J. Weber, J. Brunker and S. E. Bohndiek, *J. Nucl. Med.,* **2017**, *58*, 807-814
